# Removal of Bacteria and Organic Carbon by an Integrated Ultrafiltration—Nanofiltration Desalination Pilot Plant

**DOI:** 10.3390/membranes10090223

**Published:** 2020-09-04

**Authors:** Zahid Ur Rehman, Bayan Khojah, TorOve Leiknes, Safiya Alsogair, Mona Alsomali

**Affiliations:** 1 Water Desalination and Reuse Center (WDRC), King Abdullah University of Science and Technology (KAUST), Thuwal 23955-6900, Saudi Arabia; bayan.khojah@kaust.edu.sa (B.K.); torove.leiknes@kaust.edu.sa (T.L.); 2 DuPont Water Solutions, King Abdullah University of Science and Technology (KAUST), Thuwal 23955-6900, Saudi Arabia; safiya.sagair@gmail.com; 3 Dow Middle East Innovation Center (MEIC), King Abdullah University of Science and Technology (KAUST), Thuwal 23955-6900, Saudi Arabia; Mialsomali@dow.com

**Keywords:** ultrafiltration, nanofiltration, assimilable organic carbon, biofouling, membrane cleaning

## Abstract

Fouling caused by organic matter and bacteria remains a significant challenge for the membrane-based desalination industry. Fouling decreases the permeate quality and membrane performance and also increases energy demands. Here, we quantified the amount of organic matter and bacteria at several stages along the water-treatment train of an integrated ultrafiltration–nanofiltration seawater treatment pilot plant. We quantified the organic matter, in terms of Total Organic Carbon (TOC) and Assimilable Organic Carbon (AOC), and evaluated its composition using Liquid Chromatography for Organic Carbon Detection (LC-OCD). The bacterial cells were counted using Bactiquant. We found that ultrafiltration (UF) was effective at removing bacterial cells (99.7%) but not TOC. By contrast, nanofiltration (NF) successfully removed both TOC (95%) and bacterial cells. However, the NF permeate showed higher amounts of AOC than seawater. LC-OCD analysis suggested that the AOC was mostly composed of low molecular weight neutral substances. Furthermore, we found that the cleaning of the UF membrane using chemically enhanced backwash reduced the amount of AOC released into the UF permeate. By implementing the cleaning-in-place of the NF membrane, the pressure drop was restored to the normal level. Our results show that the UF and NF membrane cleaning regimes investigated in this study improved membrane performance. However, AOC remained the hardest-to-treat fraction of organic carbon. AOC should, therefore, be monitored closely and regularly to mitigate biofouling in downstream processes.

## 1. Introduction

Freshwater-stressed countries, such as those in the Middle East, rely heavily on energy-dependent techniques such as water desalination to produce freshwater for their domestic and agricultural needs. However, membrane-based technologies are increasingly preferred for producing freshwater from seawater, mainly because they consume less energy, and membrane performance has improved significantly, with better salt rejection, higher fluxes, and longer lifespans. However, the fouling of membranes caused by organics, inorganics, and microorganisms in seawater remains a critical problem [1].

Water quality parameters such as the Total Organic Carbon (TOC), Assimilable Organic Carbon (AOC), and bacterial concentration have been correlated with membrane biofouling [2,3]. The AOC comprises the smallest fraction of the TOC (0.1–9%) that is readily consumed by bacteria, resulting in their proliferation [4]. Organic carbon in the form of polysaccharides and proteins can act as a foulant that deposits on membranes as well as an energy source that supports the growth of microorganisms [5]. Growing microorganisms produce extracellular polymeric substances (EPS) that, in combination with other organic substances, generate foulants of varying sizes. A critical size range of foulants (1–1.2 mm) is mainly responsible for membrane fouling [6]. Furthermore, acidic polysaccharides can cross-link in the presence of cations to form transparent exopolymer particles that deposit on membranes as a gel-like layer, also contributing to membrane biofouling [7]. The biofouling of membranes leads to an increase in the demand for operational energy and reduces the quality of the permeate water [8]. Studies show that the AOC is an important parameter for estimating the biological stability and fouling potential of raw and treated water [4,9].

Different physical and chemical pretreatments are applied to remove impurities and potential foulants in raw seawater [10]. The extensive use of chemicals in pretreatment steps can negatively impact the environment; therefore, the improvement and widespread implementation of physical pretreatment methods is preferred. Low-pressure ultrafiltration (UF) membranes are gaining popularity for the pretreatment of seawater [10,11]. Studies have shown that UF performs better than conventional pretreatment methods in removing foulants and requires fewer chemicals [12]. The application of UF pretreatment has been shown to be economically feasible and advantageous [13]. However, an in-depth investigation is required on the nature of the foulants that can pass through UF membranes and cause fouling downstream, to optimize and improve UF operational conditions.

Nanofiltration (NF) membranes are commonly used in the municipal, pharmaceutical, and textile industries to remove organic and trace contaminants and produce high-quality water. However, the biofouling of NF membranes can compromise NF performance, integrity, and permeate water quality [14]. The accumulation of biomass on the membrane surface increases the pressure drop along the membrane module [15], which consequently increases the energy consumption. The mechanisms and implications of biofouling in the NF process are well studied [16,17]. However, a better understanding of the role of biocides in the removal of organic foulants, specifically AOC, is desirable. Furthermore, detailed analyses of the physical and chemical natures of organic carbon that can pass through the NF membrane are required.

This study aims to investigate the removal of biological contaminants in seawater by an integrated UF and NF membrane system. We quantify the removal of bacterial cells, AOC, and TOC from seawater by the UF and NF membranes. The size and charge characteristics of organic carbon at different stages of water treatment are also investigated using liquid chromatography for organic carbon detection (LC-OCD). Furthermore, we evaluate the efficiency of cleaning methods, such as cleaning in place (CIP) and chemically enhanced backwash (CEB), in removing contaminants and maintaining the performance of the membrane. Lastly, we investigate the use of the biocide 2,2-dibromo-3-nitrilopropionamide (DBNPA) to remove organic matter and microbes during seawater desalination treatment.

## 2. Materials and Methods

This study was conducted on a pilot-scale NF membrane-based seawater desalination plant located 120 km north of Jeddah (22.306152, 39.107633), Saudi Arabia, and 1.2 km inland from the Red Sea coast (Figure S1). The intake pipe equipped with a 2 cm mesh screen was located at a depth of 10 m below sea level and could deliver up to 1920 m^3^ of seawater per day.

Intake seawater was collected in a tank before passing through UF membranes for pretreatment. The UF permeate was collected in a tank and treated with DBNPA, sodium metabisulfite, and an antiscalant as it left the tank and entered into the NF treatment system. NF was applied for the enhanced removal of sulfate. The UF and NF membrane (DuPont Water Solutions, Edina, MN, USA) specifications are given in Table 1.

### 2.1. Sample Collection

**Figure 1:**
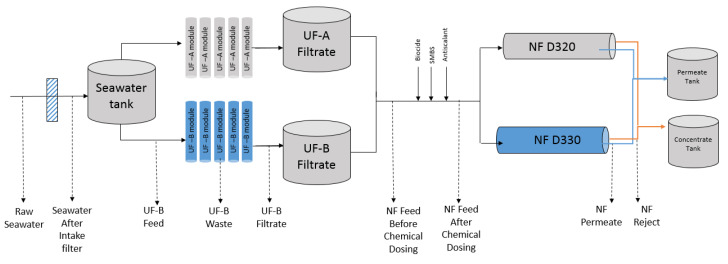
Schematic of pilot plant and sampling points.

Triplicate samples of the raw seawater, seawater after the intake filter, UF feed, UF filtrate, NF feed (before and after chemical dosing with DBNPA, Sodium metabisulphite (SMBS), and the antiscalant (these chemicals were purchased from AES Arabia Ltd., Riyadh, Saudi Arabia)), NF permeate, and NF reject were collected. Details of the sampling points and the rationale for the collection and sample processing methods are given (Table S1, Figure S5). A graphical representation of the steps followed in seawater treatment and the sampling points is shown in Figure 1. The average seawater temperature was 32 ± 1.0 °C during these sampling runs.

### 2.2. Membrane Cleaning Procedure

CEB was implemented every 24 h to clean the UF membrane. The steps involved in the CEB procedure are described in Table S2. Briefly, air scouring was first carried out to remove the foulants from the membrane, followed by draining and hydraulic cleaning. Next, the membranes were soaked with NaOCl to remove the foulants that had accumulated in the membrane pores, followed by a forward flush and normal backwash. To study the effect of CEB, we took water samples before CEB (in this case, the previous CEB occurred 22–24 h previously) and immediately after CEB was completed.

CIP was implemented for the cleaning of the NF membrane. The addition of biocide was halted around a week before CIP was performed. The steps involved in the CIP procedure are described in Table S3. Briefly, low flow recirculation was performed first. In this step, the chemical solution was pumped into the membrane under low pressure. This was followed by a soaking step for between 1 h and overnight, depending on the fouling severity. Next, high flow recirculation was performed, where the pressure was higher than that in the first stage to ensure better flushing. Lastly, a flush out using deionized water was performed to flush out any chemical residuals and dislodged particles. The plant was shut down for one or two days to implement CIP. Samples were taken three days before and three days after the implementation of CIP to study the effect of CIP.

The biocide DBNPA was dosed in the NF feed at a concentration of 20 ppm, three times a week, for three hours. The SMBS and antiscalant were dosed continuously at the concentration of 1 ppm. While DBNPA was dosed, the addition of SMBS was stopped. Samples were taken within one to two days of biocide dosing.

### 2.3. Water Quality Parameters

The pH and conductivity of all the water samples were measured using meters from Hach, Loveland, CO, USA.

### 2.4. AOC Measurement

The AOC was measured as described previously [18]. Briefly, volumes of 190 μL of samples, in triplicate, were added to 96 well plates. The samples were filtered using 0.22 μm RC filters as soon as possible after sample collection (within two hours) to remove bacteria and were stored at 4 °C until analysis. For the luminescence assay, 190 mL of glucose solution (10, 25, 50, and 100 μg-C/L) in Artificial Sea Water (ASW) was dispensed in 96-well microtiter plates (Greiner Bio-One, Frickenhausen, Germany). The wells were subsequently inoculated with 10 μL of *Vibrio fischeri* MJ-1 containing around 10^6^ Colony Forming Units (CFU). The 96-well plates were covered with an adhesive plate seal to minimize evaporation. Afterward, the plates were transferred to the Spectramax L plate reader (Molecular Devices, San Jose, CA, USA) set to 25 °C, and the luminescence was measured every 5 min for 12 h. The SoftMax^®^ Pro software v5.4 was used to analyze the data. Glucose (Sigma-Aldrich, St. Louis, CA, USA) was used as the only carbon source. The ASW without glucose was used as the negative control, while Marine Broth (MB) was used as the positive control.

Artificial Seawater (ASW) was prepared as described by Jeong et al. [18].

Briefly, 13.5 g of NaCl, 1.96 g of Na_2_SO_4_, 0.107 g of NaHCO_3_, 0.33 g of KCl, 0.053 g of KBr, 2.5 g of MgC_l2_ · 6H2O, 0.55 g of CaCl_2_ · 2H_2_O, 0.0107 g of SrCl_2_ · 6H_2_O, and 0.0107 g of H_3_BO_3_ were dissolved in 1 L of Milli-Q water. The ASW was fortified with 9.52 mM NH_4_Cl and 1.32 mM K_2_HPO_4_. The final pH was 7.5.

### 2.5. TOC Measurement

Volumes of 20 mL of the water samples were used to measure the TOC (Shimadzu, Kyoto, Japan). The TOC vials were rinsed with sample three times before being filled. The TOC standard solutions with organic carbon concentrations of 1 mg/L and 5 mg/L were also measured before, during, and after sample measurement.

### 2.6. Liquid Chromatography for Organic Carbon Detection (LC-OCD)

LC-OCD (DOC Labor, Karlsruh, Germany) analysis was performed to identify the different components of organic carbon. LC-OCD quantifies biopolymers, humic substances, building blocks, and Low Molecular Weight (LMW) neutral substances and acids. The LC-OCD columns were cleaned using 0.1 M NaOH (Sigma-Aldrich, St. Louis, CA, USA), followed by Milli-Q washing, before running the samples. A total of 4 mL of cleaning solution was injected and allowed to run for 260 min. After cleaning, 10 mL volumes of samples (already filtered through 0.45 μm filters) were injected into the size-exclusion separation column for the subsequent measurement of the dissolved organic carbon. The mobile phase for LC-OCD consisted of 7.5 g of disodium hydrogen phosphate (Honywell Fluka, Charlotte, NC, USA) and 12.5 g of potassium phosphate (Honywell Fluka, Charlotte, NC, USA) dissolved in 5 L of Milli-Q water. A sample volume of 2 mL was injected and allowed to run for 180 min.

The chromatograms produced by LC-OCD were integrated and analyzed using ChromCALC (DOC Labor, Karlsruh, Germany).

### 2.7. Quantification of Bacteria

The quantification of the bacteria in the samples was performed using Bactiquant (Mycometer, Tampa, FL, USA). A volume of 250 mL of seawater (500 mL of treated samples) was filtered through 0.45 μm filters (Millipore, Burlington, MA, USA) to harvest the bacteria. The filters were incubated with an artificial substrate provided with the kit for 60 min, and the fluorescence was measured. The results for the fluorescence are given in BQV (Bactiquant value) units, which represent fluorescence.

## 3. Results and Discussion

### 3.1. Physical Parameters

Physical parameters such as the pH and conductivity were measured in water samples collected at different stages of the water treatment train (Figure S2). The pH of the raw seawater was 8.1 ± 0.03, which remained constant until chemical dosing by antiscalant, DBNPA, and SMBS, which resulted in the reduction of the pH to 7.8 ± 0.05. The treatment through the NF membrane resulted in a further slight reduction in pH to 7.6 ± 0.03. The conductivity of the raw seawater was 62 ± 0.16 mS/cm^2^. The treatment of the water with the UF membrane and the addition of chemicals did not change the conductivity (Figure S2). Treatment with NF resulted in a reduction in the conductivity by ~20% to 49.6 ± 0.3, while the conductivity of the NF reject increased to 72.5 ± 0.3. The reduction in conductivity can be attributed to the rejection of the multivalent ion by the NF membrane, whereas monovalent ions can pass through the NF membrane. Our results are in agreement with previous reports suggesting that NF membranes can retain 79–89% of the electrical conductivity [19]. The NF membranes have a bigger pore size; therefore, they are not as effective as reverse osmosis (RO) at removing salts from seawater.

### 3.2. UF Membranes Remove Bacteria Effectively

**Figure 2:**
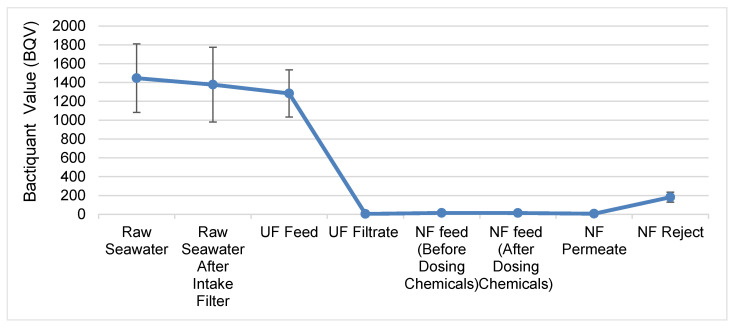
Removal of bacteria in the UF–NF treatment train. The stages of water treatment are given along the *X*-axis, while the Bactiquant values as BQV units are given along the *Y*-axis. The values represent the averages of five measurements conducted over three weeks. Error bars represent standard deviations.

The UF membrane successfully removed ~99.7% of the bacterial cells from the UF feed (Figure 2). These results were not unexpected, as UF membranes are known for their ability to filter bacterial cells effectively [20]. After UF treatment, the number of bacterial cells remained remarkably low in all of the downstream seawater treatment steps. The final NF permeate had ~99.99% fewer bacteria compared to the raw seawater (Figure 2). Nevertheless, some bacterial cells were still detected in the NF permeate. Other studies have shown that some bacterial cells are capable of passing through theoretically impenetrable RO membranes [21]. If left untreated, these bacterial cells can grow in the presence of nutrients and form problematic biofilms in the water distribution system. Therefore, specific measures should be taken to prevent biofouling in the downstream water distribution system.

### 3.3. NF Membrane Removes TOC from Water More Effectively

**Figure 3:**
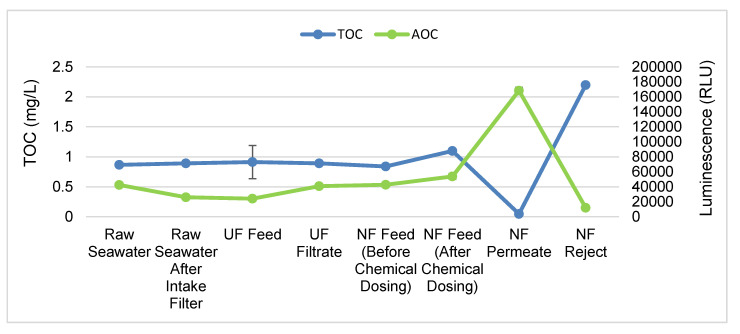
Assimilable Organic Carbon (AOC) and Total Organic Carbon (TOC) trends in the UF–NF treatment train. The different stages of treatment are given along the *X*-axis, and the TOC and luminescence (relative light units) are given along the *Y*-axis. The results represent the averages of five measurements performed over three weeks. Error bars denote standard deviations.

At the time of conducting these experiments, the TOC of the samples taken from the Red Sea was 0.9 ± 0.03 mg/L, which is in line with that in previous studies conducted in the Red Sea [22]. We did not observe any difference in the concentration of TOC in the UF feed and permeate. Interestingly, the addition of chemicals (SMBS and antiscalant) caused a 26% increase in TOC (Figure 3). This increase in TOC is most likely caused by the antiscalant, as SMBS is an inorganic compound and thus cannot contribute to the organic carbon concentration. Although the exact chemical nature of the antiscalant used in this study is unknown, antiscalant has been shown, however, to increase organic fouling [23]. Furthermore, antiscalants, such as polyacrylates and carboxylated dendrimeric polymers, contain carbon-containing functional groups (carboxylic acid and methyl groups, for example), which may lead to increased TOC in the water.

Our results show that UF membranes are not effective at removing organic carbon (Figure 3), in line with previous studies that indicated that UF membranes are not effective at removing natural organic substances [12,24]. Therefore, the use of adsorbents or coagulants for the removal of organic material prior to UF treatment has been recommended. Adsorbents such as powdered activated carbon (PAC) and granular activated carbon have been shown to effectively remove organic contaminants and reduce the irreversible fouling of UF membranes [25,26]. More recently, the use of carbon nanotubes as adsorbents in water/wastewater treatment is gaining attention due to their superior capacity to remove organic substances and microorganisms [27].

By contrast, NF membranes are effective at removing organic carbon, reducing the organic carbon by 95% relative to that in the NF feed (Figure 3). Our results are in agreement with those of previous studies that have outlined the effective removal of organic carbon by NF membranes. Furthermore, previous studies have indicated that the removed organic carbon mostly belongs to high molecular weight compounds such as polysaccharides and humic substances [16]. The organic carbon rejected by the NF is released into the NF reject, which showed an almost 98% increase in TOC compared to the NF permeate.

### 3.4. Treatment with UF and NF Membranes Leads to Increase in AOC in the Permeate

In this experiment, we used luminescence as an indirect measure of the amount of AOC in the water samples. The average luminescence caused by the growth of *V. fischeri* in the samples from different treatment steps is shown in Figure 3. We observed a 30% decrease in the luminescence in water samples collected after the intake filter relative to in the raw seawater. The intake filter pore size was 250 μm, which is too large to filter out organics and AOC. We hypothesize that the biofilm growing on the intake filter consumed the AOC present in raw seawater. At the same time, we would expect that the biofilm on the intake filter would also release EPS or cellular debris as a consequence of the bacterial growth. However, we detected no increase in TOC after the intake filter (Figure 3). Therefore, further exploration of the effect of the seawater intake screen on the concentration of AOC is necessary.

In contrast to that with the intake filter, the treatment of seawater with the UF membrane resulted in a 66% increase in luminescence from *V. fischeri*, indicating an increase in AOC (Figure 3). The increased AOC in the UF filtrate could be the result of the growth and lysis of microbial cells deposited on the UF membrane. Another reason, as suggested previously, could be the hydrolysis of the organic matter deposited on the UF membranes, leading to an increase in the AOC [28]. Another study has shown that as the amount of organics before the granular activated carbon (GAC) bed filter increases, the amount of AOC released at the outlet also increases [29]. These results suggest that the regular cleaning of the UF membrane is paramount for reducing the amount of AOC in the UF filtrate. To confirm this hypothesis, we measured AOC in the UF permeate after CEB treatment, and our results showed that the AOC decreased by almost 50% (Figure S3).

The addition of SMBS and antiscalant led to a 29% increase in luminescence (Figure 3). This increase could be the result of the choice of antiscalant, which releases nutrients and organics that the bacteria can feed on [23]. Moreover, the neutralization of chlorine with SMBS possibly generated favorable conditions for bacteria to grow.

Similar to the UF, treatment with NF resulted in an increase in luminescence (169%) compared to that in the NF feed. Although the exact reason for this increase in AOC is unknown, it could be the result of microbial growth and lysis on the NF membrane or the hydrolysis of organic matter generating LMW organic matter. Indeed, studies have shown that LMW neutral substances have higher permeability through NF and RO membranes due to the lack of electrostatic repulsion [16,30]. These results suggest that other treatment options such as biological activated carbon filters (BACF) should be employed for the removal of AOC [31]. The NF reject showed a roughly 90% reduction in luminescence compared to the NF feed. This decrease in luminescence may not necessarily represent a decrease in AOC because higher concentrations of salt in the NF reject may be unsuitable for the growth of *V. fischeri*.

### 3.5. CEB of UF Membrane Decreases AOC in UF Permeate

The CEB showed no effect on the concentrations of TOC and bacterial cells; these parameters remained similar before and after CEB (Figure S3a,b). The higher frequency (every 24 h) of CEB application in this study possibly masked any CEB effect. An increase in the duration between successive CEB treatments might reveal the effect of CEB. Nevertheless, CEB reduced the amount of AOC released into the UF filtrate, as indicated by the decreased luminescence from *V. fischeri* (Figure S3c). These results suggest that CEB reduced the biomass accumulated on the UF membranes, resulting in a reduced release of AOC into the UF permeate. Our results support the findings from previous studies that biomass that accumulated on the UF membrane contributed to an increase in the AOC in the UF filtrate (Figure 3) [28,29]. Furthermore, the reduced amount of AOC after the UF membrane was not caused by any residual chlorine because the amount of AOC remained constant even after the addition of SMBS, which neutralizes chlorine (Figure S3c). These results show that the regular cleaning of the UF membrane is required to reduce AOC, which could have a negative impact on downstream operations.

### 3.6. Biocide DBNPA Is Effective at Killing Bacterial Cells in NF Feed

We evaluated the effect of the biocide DBNPA on organics and microbial cells downstream of the UF treatment. Dosing the NF feed with DBNPA three times a week for three hours caused a 50% reduction in bacterial cells in the NF feed and NF reject (Figure S4a). The bacterial cells were very low in the NF permeate with or without DBNPA.

The biocide addition did not have any impact on the TOC. The TOC remained constant (very low) in the NF feed and permeate (Figure S4b). We observed a slight increase in the TOC (9%) in the NF reject in the absence of biocide, which could be attributed to increased bacterial growth and EPS production on the NF membrane.

We found the overall trend of the AOC to be similar in the presence and absence of the biocide DBNPA. Thus, the AOC was elevated in the NF feed and NF permeate after the addition of chemicals (antiscalant and SMBS), whereas it was reduced in the NF reject, irrespective of dosing with DBNPA (Figure S4c).

**Figure 4:**
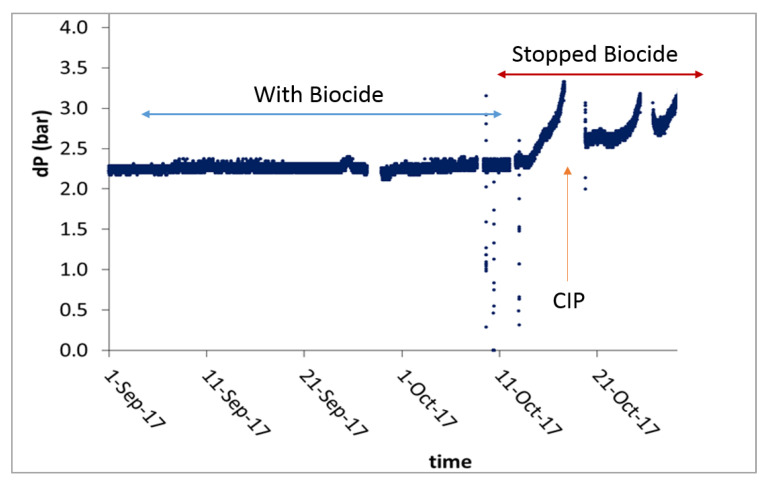
Pressure drop in the NF membrane system with and without biocide (2,2-dibromo-3-nitrilopropionamide (DBNPA)) dosing and application of cleaning in place (CIP).

Although the addition of DBNPA did not affect the amount of organic matter in the NF feed and the permeate, the impact on the membrane performance was notable. Our results showed that, when DBNPA was added, the pressure drop across the NF modules remained constant (2.3 bar) for six weeks (Figure 4). However, when the dosing of DBNPA was ceased, the pressure drop increased to 3.5 bar in less than two weeks (Figure 4). These results suggest that an increase in pressure drop is most likely due to biofouling because, in the absence of biocide, the microbes in the NF feed will grow and establish a biofilm on the NF membrane. The increase in pressure drop required us to perform CIP, which restored the system’s performance and reduced the pressure drop to ~2.5 bar (Figure 4). Our results support a previous study showing that DBNPA is effective in preventing biofilm formation on membranes. However, once the biomass has accumulated, DBNPA cannot be used for curative biofouling control [32]. Therefore, CIP was performed to restore the membrane’s performance.

### 3.7. LC-OCD Analysis Suggests AOC Is Primarily Composed of LMW Neutral Substances

**Figure 5:**
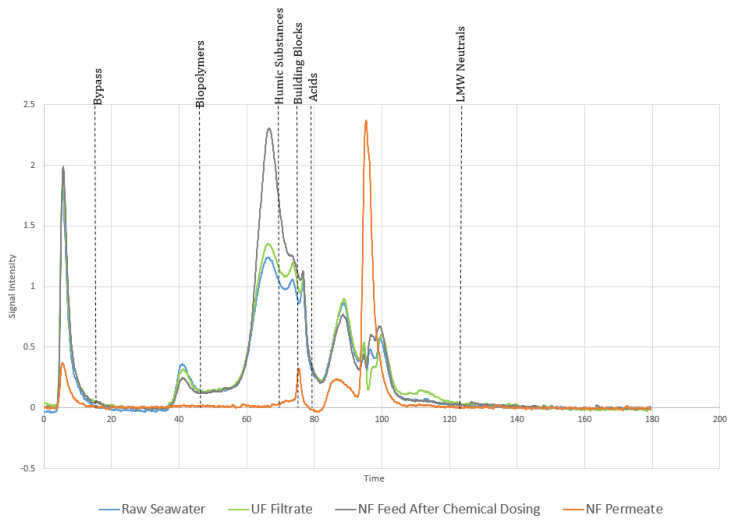
Liquid Chromatography for Organic Carbon Detection (LC-OCD) Chromatogram showing the composition of the organic substances in raw seawater, the UF filtrate, the NF feed after chemical dosing, and the NF permeate.

To better understand the composition of the organics in the water samples collected at different stages of treatment, we performed LC-OCD analysis. Given that the AOC increased to 169% in the NF permeate, we wished to determine the fraction of organic carbon that constitutes the AOC. The LC-OCD analysis separated the organic compounds based on their molecular weights and provided information on their chemical nature. The LC-OCD chromatogram in Figure 5 shows that the organic content of raw seawater and UF treated seawater was very similar. However, a slight increase (10%) was detected in humic-like substances and building blocks in the UF filtrate (Figure 5). The addition of chemicals (SMBS and antiscalant) to the NF feed resulted in a 100% increase in humic-like substances. Based on previous studies, it is most likely that the antiscalant contributed to humic-like substances, which increased the TOC in the NF feed (Figure 5) [23]. These findings highlight the need to consider the choice of antiscalant carefully.

Our results further show that the NF can effectively remove high molecular weight organic substances. For instance, on average, 95% and 94% of the biopolymers and humic substances were removed, respectively. By contrast, building blocks were reduced by 23% (Figure 5). Interestingly, the NF treatment led to an increase in LMW neutral substances in the permeate. These LMW neutral substances are composed of organics, such as simple sugars, which can lead to biofilm formation [16]. Overall, our results are consistent with a previous study showing that NF can effectively remove biopolymers, humic substances, and charged LMW organics, whereas LMW neutral substances can still pass through. Contrary to our results, Maylan et al. showed that the AOC in NF treated water was lower than that in raw water [16]. These differences in AOC removal can be attributed to differences in membrane characteristics and the nominal pore sizes of the NF membranes used. Alternatively, the observed differences could be attributed to differences in the NF process configurations and AOC assay.

Nevertheless, our results showed that most of the dissolved organic carbon (DOC) in the NF permeate constitutes LMW neutral substances and a small amount of LMW acids (Figure 5). Previous studies have shown that LMW organic acids (at least the tested ones) were not true representatives of AOC [16,33]. Therefore, based on the higher abundance of LMW neutral substances and AOC in the NF permeate, it appears that these two components are related. Our results, therefore, support previous suggestions that LMW neutral substances significantly contribute to AOC concentrations [16]. However, the contribution of naturally occurring LMW acids to AOC needs to carefully evaluated.

## 4. Conclusions

In this study, we evaluated the effect of pretreatment methods, such as the use of UF membranes and the chemical dosing of antiscalant and biocides, on the quality of NF feeds and permeates. Furthermore, we investigated the effect of cleaning methods, such as CEB and CIP, on the removal of organics to restore the NF membrane’s performance. We found that:

- UF membranes are effective at removing bacterial cells but not organics.
- The antiscalant contributes humic-like organic substances to the water.
- The NF membrane used in this study effectively removes bacteria and TOC but not AOC. We found an increased AOC concentration in the NF permeate.
- LMW neutral sustances constitute a significant component of AOC.
- The use of the biocide DBNPA and CIP improves the performance of the NF membrane by delaying the pressure drop.

Therefore, we conclude that UF–NF pretreatment technologies are not an effective solution for removing AOC. Furthermore, if NF treatment aims to produce potable water, the concentration of AOC in the NF permeate should be continuously monitored, and measures should be taken to reduce biofilm formation in the water distribution system. The application of BACF should be explored for the effective removal of AOC. Moreover, although antiscalants are very effective at controlling inorganic fouling, they can nevertheless contribute to organic fouling. Therefore, the chemical composition and concentration of antiscalant should be carefully considered.

## Figures and Tables

**Table 1:** Specifications of ultrafiltration (UF) and nanofiltration (NF) membranes.

	Ultrafiltration Membrane	Nanofiltration Membrane
Membrane Type	Polyvinylidene fluoride(PVDF)	Thin-film composite
Nominal Pore Diameter (nm)	30	1
Surface area (m^2^)	77	40.9
Average Flux (LMH)	78	28
Feed flow (m^3^/h)	30	13.74
System Pressure (bar)	13–18	4–6

## Supplementary Materials

The following are available online at https://www.mdpi.com/2077-0375/10/9/223/s1. Figure S1: Location of Dow nanofiltration pilot plant. Figure S2: Physical parameters of seawater in the UF–NF treatment train. The pH and conductivity are given along the *Y*-axis, while the sampling location is along the *X*-axis. The values are the averages of five measurements over three weeks. Error bars represent standard deviations. Figure S3: Effect of CEB on bacteria, TOC, and AOC at stages downstream of UF. The results shown are the averages of five independent samples collected over three weeks. Error bars represent standard deviations. Figure S4. Effect of a biocide (DBNPA) on bacteria (**A**), TOC (**B**), and AOC (**C**) removal by NF. The results here show the average of two sampling runs. Error bars indicate standard deviations. Figure S5. Flowchart showing sample processing after collection. Table S1: Sample collection points and rationale. Table S2. Steps followed during chemically enhanced backwashing of the UF. Table S3. Steps followed during cleaning for the NF membrane.
