# Supplementary material for: Removal of Bacteria and Organic Carbon by an Integrated Ultrafiltration—Nanofiltration Desalination Pilot Plant
Source: Membranes (Basel). 2020 Sep 4;10(9):223. doi: 10.3390/membranes10090223 (PMC7559365; doi:10.3390/membranes10090223)
Supplement: Supplementary file 1 [file membranes-10-00223-s001.pdf]

Supplementary material

Removal of bacteria and organic carbon by an Integrated Ultrafiltration - Nanofiltration Desalination Pilot Plant

Zahid Ur Rehman[a*#], Bayan Khojah[a#,],TorOve Leiknes[a]

Category: Original research paper

[a]Water Desalination and Reuse Center (WDRC), King Abdullah University of Science and Technology (KAUST), Thuwal 23955-6900, Kingdom of Saudi Arabia

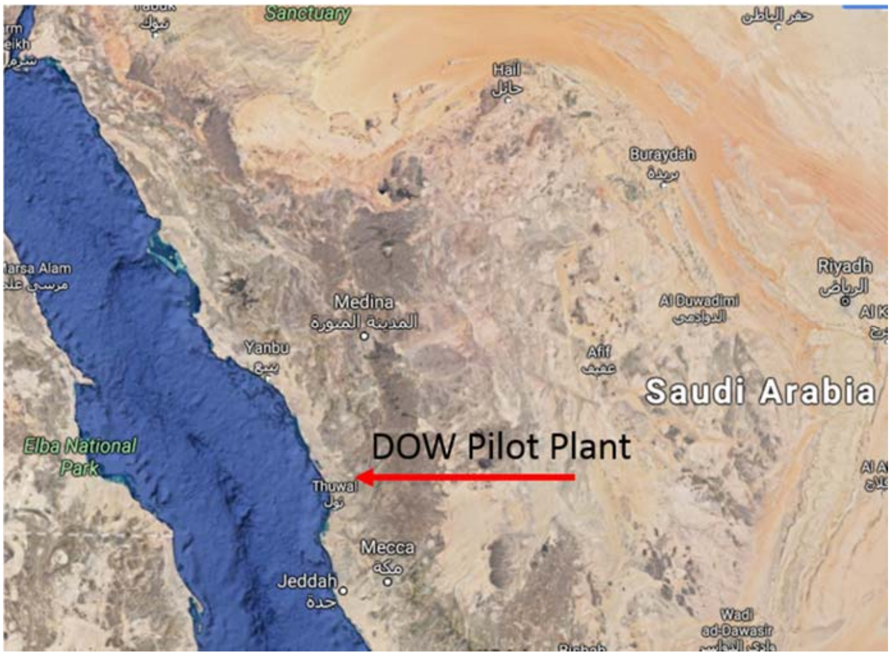

Figure S1: Location of Dow nanofiltration pilot plant

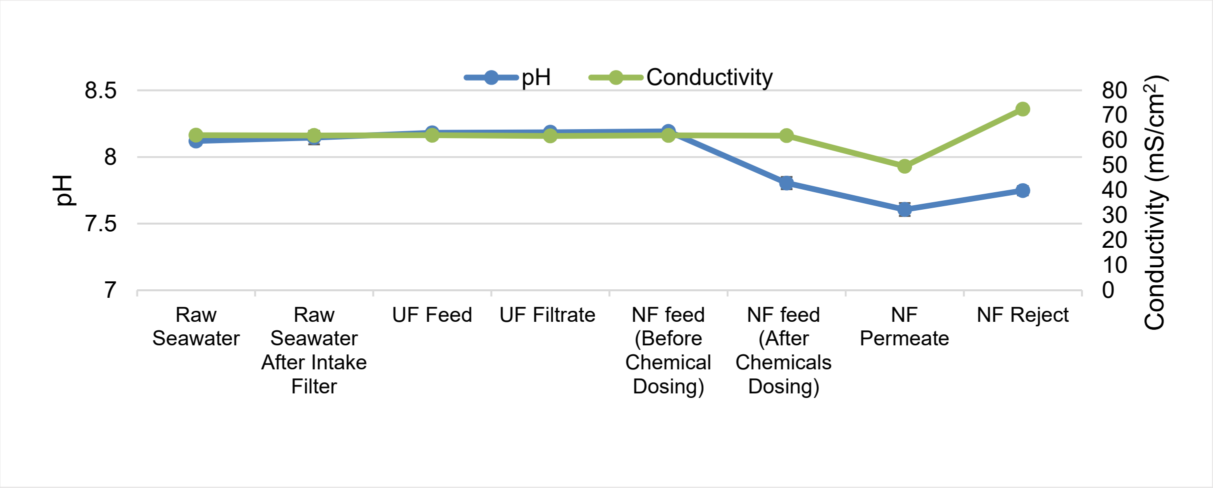

Figure S2: Physical parameters of seawater in the UF-NF treatment train. The pH and conductivity are given along the Y-axis, while the sampling location is along the X-axis. The values are averages of five measurements over three weeks. Error bars represent standard deviation.

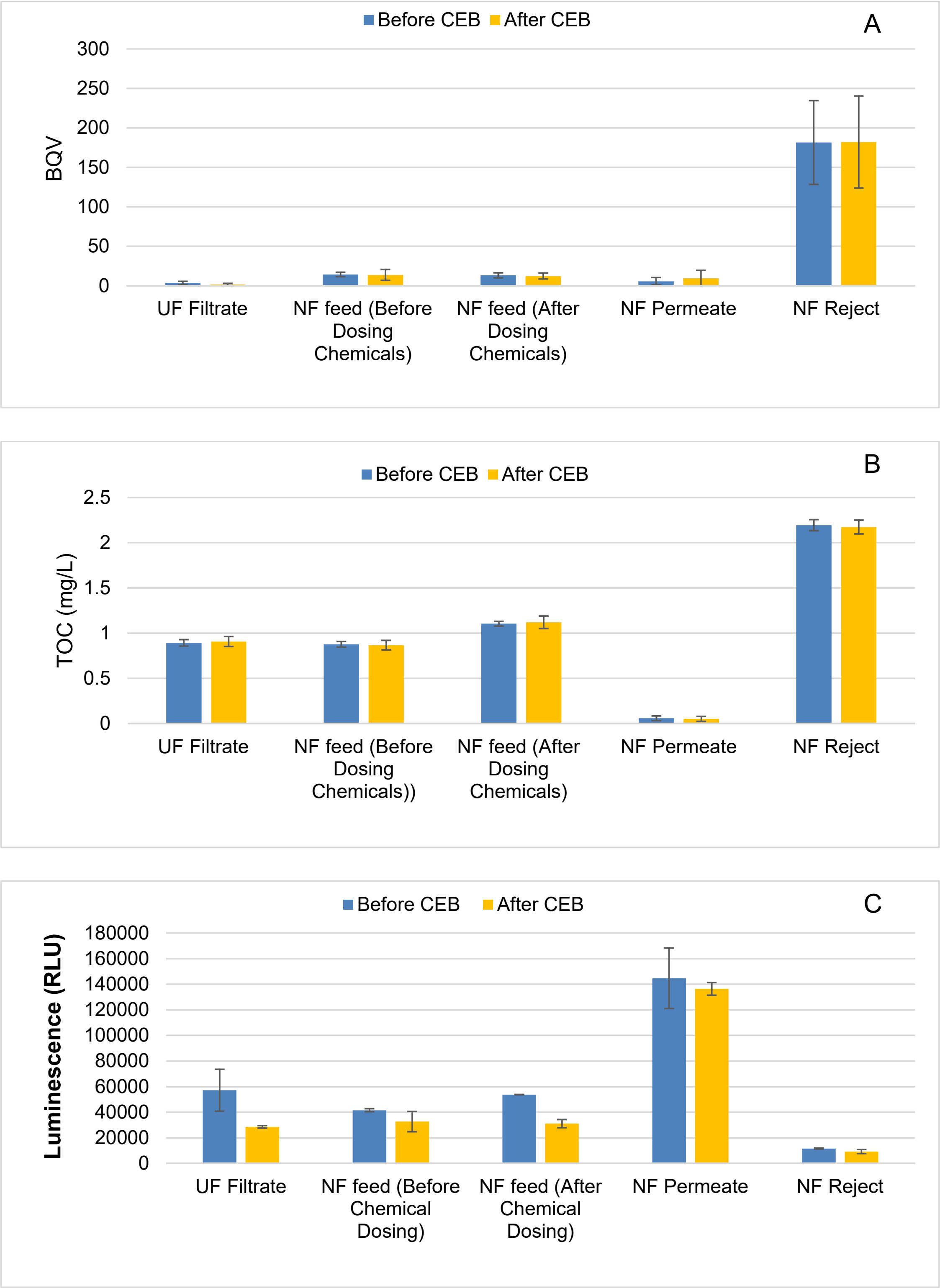
A
Before CEB
After CEB
BQV
300
250
200
150
100
50
0
UF Filtrate
NF feed (Before Dosing Chemicals)
NF feed (After Dosing Chemicals)
NF Permeate
NF Reject
B
Before CEB
After CEB
TOC (mg/L)
2.5
2
1.5
1
0.5
0
UF Filtrate
NF feed (Before Dosing Chemicals))
NF feed (After Dosing Chemicals)
NF Permeate
NF Reject
C
Before CEB
After CEB
Luminescence (RLU)
180000
160000
140000
120000
100000
80000
60000
40000
20000
0
UF Filtrate
NF feed (Before Chemical Dosing)
NF feed (After Chemical Dosing)
NF Permeate
NF Reject

Figure S3: Effect of CEB on bacteria, TOC, and AOC at stages downstream of UF. The results shown are the average of five independent samples collected over three weeks. Error bars represent standard deviation. Table S1: Sample collection points and rationale.

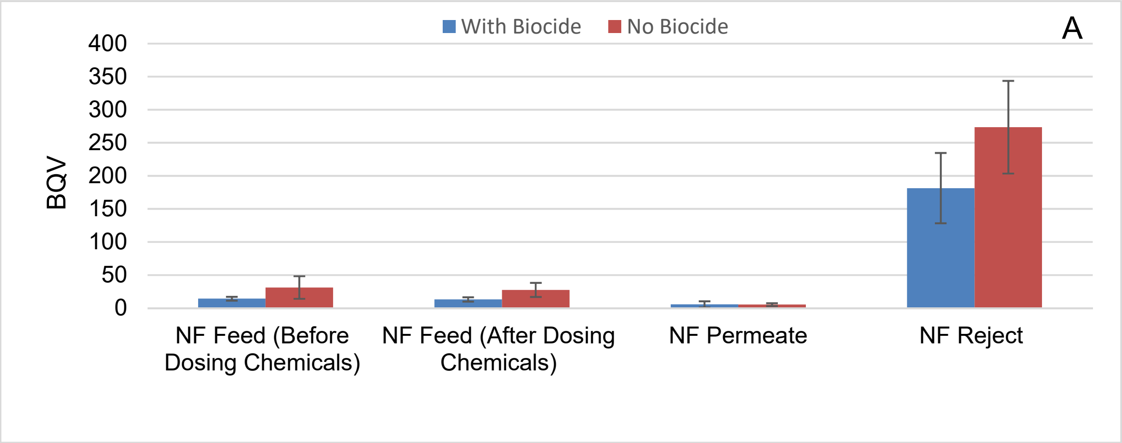
With Biocide
No Biocide
A
BQV
400
350
300
250
200
150
100
50
0
NF Feed (Before Dosing Chemicals)
NF Feed (After Dosing Chemicals)
NF Permeate
NF Reject

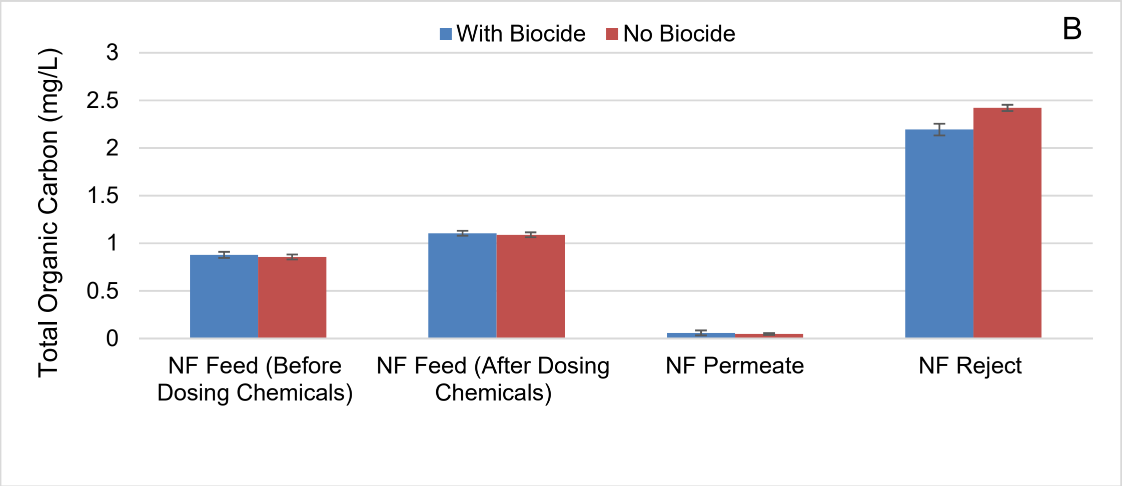
With Biocide
No Biocide
B
Total Organic Carbon (mg/L)
3
2.5
2
1.5
1
0.5
0
NF Feed (Before Dosing Chemicals)
NF Feed (After Dosing Chemicals)
NF Permeate
NF Reject

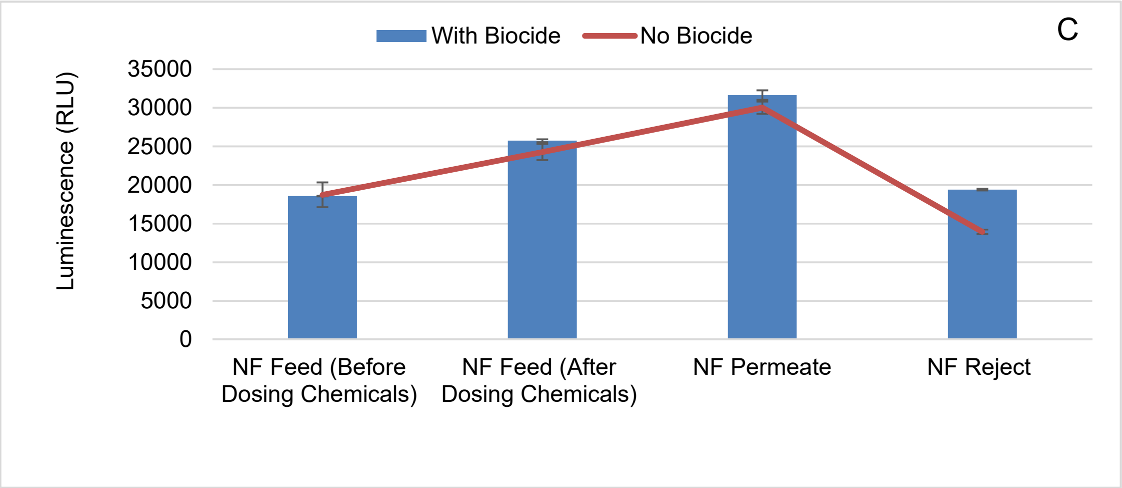

Figure S4. Effect of biocide (DBNPA) on bacteria (A), TOC (B) and AOC (C) removal by NF. The results here show the average of two sampling runs. Error bars represent standard deviation.

Table S1. Sample collection points and rational.

<table>
<tr><th>#</th><th>Time of collection</th><th>Sample ID</th><th>Collection Rational</th></tr>
<tr><td>1</td><td rowspan="3">Before CEB</td><td>Raw Seawater</td><td>To check the seawater characteristics before any treatment or filtration</td></tr>
<tr><td>2</td><td>Raw Seawater After Intake Filter</td><td>To test the effect of the intake filter</td></tr>
<tr><td>3</td><td>UF-B Feed</td><td>To test the specific feed for UF-B</td></tr>
<tr><td>4</td><td rowspan="5">Before CEB</td><td>UF-B Filtrate</td><td>To check the efficiency of UF-B filtration</td></tr>
<tr><td>5</td><td>NF Feed (Before Chemical Dosing)</td><td>To test the merged UF filtrate from UF B and UF A before dosing any chemicals</td></tr>
<tr><td>6</td><td>NF Feed (After Chemical Dosing)</td><td>To check how dosing the biocide, antiscalant, and SMBS effect the NF feed</td></tr>
<tr><td>7</td><td>NF Permeate</td><td>To check the efficiency of NF</td></tr>
<tr><td>8</td><td>NF Reject</td><td>To check the organic composition of the NF reject</td></tr>
<tr><td>9</td><td rowspan="5">After CEB</td><td>UF-B Filtrate</td><td rowspan="5">To test how CEB can affect the UF filtrate quality and the downstream desalination processes
*Only in phase 1</td></tr>
<tr><td>10</td><td>NF Feed (Before Chemical Dosing)</td></tr>
<tr><td>11</td><td>NF Feed (After Chemicals Dosing)</td></tr>
<tr><td>12</td><td>NF Permeate</td></tr>
<tr><td>13</td><td>NF Reject</td></tr>
</table>

Figure S5. Flowchart showing sample processing after collection.

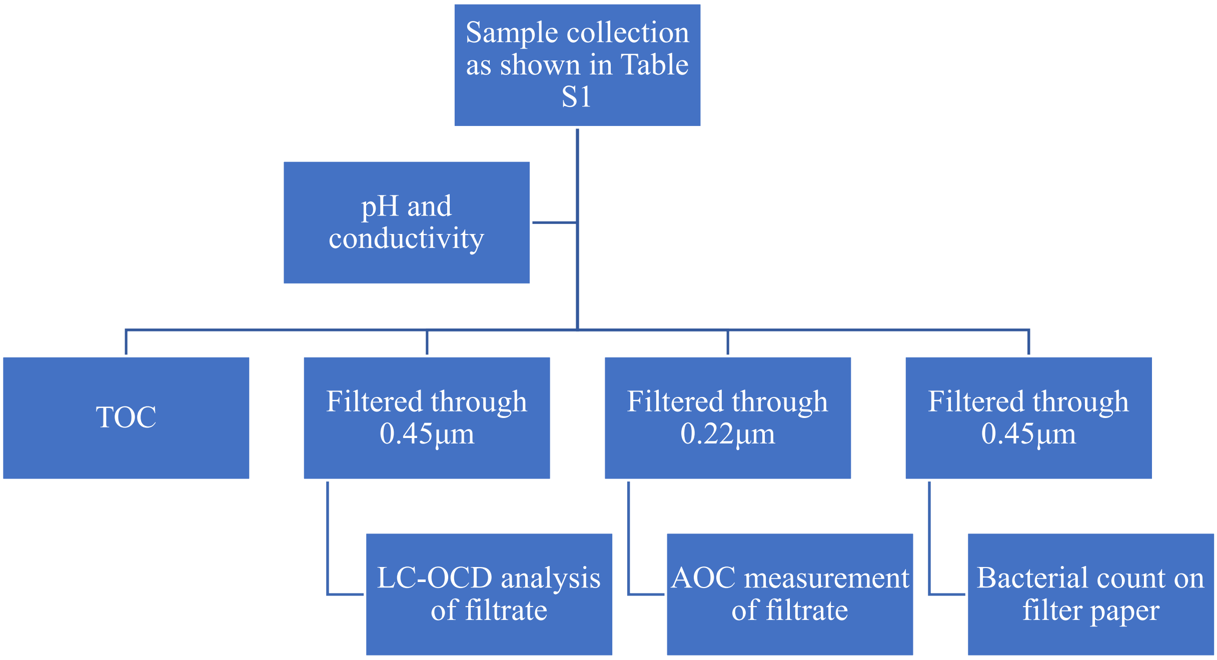

Table S2: Steps followed during chemically enhanced backwashing of the UF.

**ID**	**Step**	**Duration (s)**	**Notes**
1	Air scour	20	
2	Draining	20	
3	Hydraulic cleaning	60	
4	Chemical cleaning	120	NaOCl dosed at 350 mg/L
5	Soaking	600	
6	Forward flush	300	
7	Normal Backwash	160	

Table S3: Steps followed during cleaning for the NF membrane.

**Steps**	**Day 1: CIP alkali (1000 mg/L NaOH)**<br><br>**pH: 11**	**Day 2: CIP acid (2000 mg/L HCl)**<br><br>**pH:2.5**
1	Low flow recirculation	Low flow recirculation
2	Recycle	Recycle
3	Soaking	Soaking
4	High flow recirculation	High flow recirculation
5	NF Flush out	NF Flush out
